# 

**Published:** 1918-02-02

**Authors:** 


### CONTENTS.

PAGE

**Treatment of Disabled Sailors and Soldiers** .. 363

**Tetanus in Civilian Practice** ... ... ... 364

**Hospital and Institutional News** ... ... ... 365

The King and the Hospital Saturday Fund—The late Sir John Wolfe-Barry, K.C.B.—A Native Labour General Hospital—An Unique Christmas Card—The late Sir G. H. Philipson—The Need for Extension at Coventry—War Diet and Public Health—The Garrett Anderson Memorial—A Very Rare Distinction—Free Bread for Belfast Hospitals—The Fate of an Art Gallery—Charitable Donations and the Excess Profits Tax—Endowed Bed for an Athlete—War Bonds and Hospitals—Women as Sanatoria Superintendents—Ophthalmia Neonatorum—The Prisoners' New Paper—This Week's Drug Market.

**Some Subjective Evidences of Disease** ... 369

Myalgia: a Clinical Study.

**Lord Lister** ... ... ... ... ... ... 371

II. The Lessons of his Early Life.

**Parliament and the Profession** ... ... ... 372

**Recently Published Parliamentary Returns, etc.** 372

**The Late Sir J. Wolfe-Barry, K.C.B.** ... ... 373

An Interesting Man and a Lovable Personality.

**Massage: A Branch of Physical Treatment...** 375

Its Principles and its Value.

PAGE

**The Institutional Library** ... ... ... ... 376

Handbook of Physiology—The Pasteurisation of Milk from the Practical View-Point—Lectures on Medicine: a Handbook for Nurses—Electro-Therapeutics for Military Hospitals—The Venereal Diseases Problem—Outlines of Nursing History—The Ideal Nurse—British Boys: Their Training and Prospects—Low's Handbook to the Charities of London, 1917.

**The Matrons' and Sisters' Department** ... 379

Nursing Progress and its Developments: VII. The Probationer's Third Year.

Round the Hospitals.

Lord Derby and Mr. Smallwood—Poem: "In Flanders' Fields"—Nurse Training schools and the College—St. Thomas's Hospital Guild—Higher Salaries at Bedford.

**Some Hospital Types of British Nursing** ... 381

V. Sister Children's.

**Some Problems of To-Day**... ... ... ... 383

Rations and Health.

**The Voluntary Hospitals** ... ... ... ... 384

Treatment of Disabled Sailors and Soldiers.

**The Editor's Letter-Box** ... ... ... ... iv

The British Hospitals Association and the Treatment of Disabled Sailors and Soldiers.

**The London County and Westminster Bank, Ltd.** vi

**The Institutional Worker** ... ... (Suppl.) 1

Milk Supply and Distribution.

